# Non-alcoholic fatty liver disease (NAFLD) as a neglected metabolic companion of psychiatric disorders: common pathways and future approaches

**DOI:** 10.1186/s12916-020-01713-8

**Published:** 2020-10-01

**Authors:** Óscar Soto-Angona, Gerard Anmella, María José Valdés-Florido, Nieves De Uribe-Viloria, Andre F. Carvalho, Brenda W. J. H. Penninx, Michael Berk

**Affiliations:** 1 Department of Psychiatry, Vall d’Hebron University Hospital, Passeig de la Vall d’Hebron, 119-129, 08035 Barcelona, Catalonia Spain; 2 Deakin University, IMPACT, The Institute for Mental and Physical Health and Clinical Translation, School of Medicine, Barwon Health, Geelong, Australia; 3 Bipolar and Depressive Disorders Unit, Institute of Neuroscience, Hospital Clinic, University of Barcelona, IDIBAPS, CIBERSAM, 170 Villarroel st, 12-0, 08036 Barcelona, Catalonia Spain; 4 UGC Salud Mental, Hospital Universitario Virgen Macarena, Seville, Spain; 5 Department of Psychiatry, Hospital Clínico Universitario de Valladolid, Castilla y León, Spain; 6 Department of Psychiatry, University of Toronto, Toronto, ON Canada; 7 Centre for Addiction and Mental Health (CAMH), Toronto, ON Canada; 8 Department of Psychiatry, Amsterdam Public Health and Amsterdam Neuroscience, Amsterdam University Medical Center/Vrije Universiteit & GGZinGeest, Amsterdam, the Netherlands; 9 Orygen, The National Centre of Excellence in Youth Mental Health, the Department of Psychiatry, and the Florey Institute of Neuroscience and Mental Health, The University of Melbourne, Parkville, Australia

**Keywords:** Non-alcoholic fatty liver disease, Metabolic syndrome, Mental disorders, Psychiatry, Non-alcoholic steatohepatitis, Non-communicable disorders, Lifestyle, Inflammation, Oxidative stress, Mitochondrial

## Abstract

**Background:**

Non-alcoholic fatty liver disease (NAFLD) is characterized by hepatic steatosis in over 5% of the parenchyma in the absence of excessive alcohol consumption. It is more prevalent in patients with diverse mental disorders, being part of the comorbidity driving loss of life expectancy and quality of life, yet remains a neglected entity. NAFLD can progress to non-alcoholic steatohepatitis (NASH) and increases the risk for cirrhosis and hepatic carcinoma. Both NAFLD and mental disorders share pathophysiological pathways, and also present a complex, bidirectional relationship with the metabolic syndrome (MetS) and related cardiometabolic diseases.

**Main text:**

This review compares the demographic data on NAFLD and NASH among the global population and the psychiatric population, finding differences that suggest a higher incidence of this disease among the latter. It also analyzes the link between NAFLD and psychiatric disorders, looking into common pathophysiological pathways, such as metabolic, genetic, and lifestyle factors. Finally, possible treatments, tailored approaches, and future research directions are suggested.

**Conclusion:**

NAFLD is part of a complex system of mental and non-communicable somatic disorders with a common pathogenesis, based on shared lifestyle and environmental risks, mediated by dysregulation of inflammation, oxidative stress pathways, and mitochondrial function. The recognition of the prevalent comorbidity between NAFLD and mental disorders is required to inform clinical practice and develop novel interventions to prevent and treat these complex and interacting disorders.

## Background

Psychiatric patients suffer from a substantial reduction (ranging between 7 and 20 years) in life expectancy, with high rates of all-cause mortality [1]. It is estimated that around 60% of this excess of mortality is due to medical comorbidities, especially cardiovascular diseases [2]. In this regard, metabolic syndrome (MetS), defined as the combination of abdominal obesity, high blood pressure, low high-density lipoprotein cholesterol, elevated triglycerides, and hyperglycemia, plays an important role, since it is a major risk factor for the development of diabetes and cardiovascular disease [3]. A significant association exists between major psychiatric disorders, such as bipolar disorder, depressive disorder or schizophrenia, and MetS, partly due to psychotropic medication use (e.g., atypical antipsychotics) and an unhealthy lifestyle [4]. In a worldwide survey conducted among more than 47.000 individuals across 17 countries, specific mental disorders were associated with 1.5–13.3% of physical condition onsets [5]. Recently, in a Danish cohort of 5.9 million people, there was a median hazard ratio of 1.37 of developing a medical condition if a psychiatric disorder was present [6].

In this context, non-alcoholic fatty liver disease (NAFLD) is an entity characterized by excessive hepatic fat accumulation. This is defined as the presence of steatosis in > 5% of hepatocytes according to histological analysis [7, 8]. It is associated with insulin resistance and it was considered to be the hepatic manifestation of the MetS. Recent evidence suggests a complex, bidirectional relationship between NAFLD and cardiometabolic diseases [9].

The distinction of NASH from simple steatosis is key. Steatosis due to secondary causes, such as viruses, autoimmune responses, metabolic or hereditary factors, and drugs or toxins, should be ruled out (Table 1). Classically, the diagnosis of NAFLD required the exclusion of alcohol abuse (daily alcohol consumption greater than 30 g for men and 20 g for women) along with secondary causes for NAFLD (viral pathologies, steatogenic medications, or other monogenic hereditary disorders). However, the rising prevalence of NAFLD makes its coexistence with other chronic liver diseases quite possible. Therefore, a positive diagnosis rather than a diagnosis based on exclusion of concomitant diseases has been proposed [11], and a dual etiology for fatty liver disease is considered possible and even frequent [12].

**Table 1:** Causes of Fatty liver disease related and unrelated to patients with psychiatric disorders [10, 11]

	Related to psychiatric disorder	Non-related to psychiatric disorders
Nutritional^†^	Starvation Protein-calorie malnutrition Rapid weight loss Total parenteral nutrition Gastrointestinal surgery for obesity	
Drug-induced^‡^	Valproic acid Cocaine Glucocorticoids Antiviral agents: Zidovudine, Didanosine, Fialuridine	Synthetic estrogens Aspirin Calcium-channel blockers Tamoxifen Tetracycline Amiodarone Methotrexate Perhexiline maleate
Metabolic or genetic		Lipodystrophy Dysbetalipoproteinemia Weber–Christian disease Wolman’s disease Cholesterol ester or Glycogen storage disease Acute fatty liver of pregnancy Lysosomal acid lipase deficiency Familial combined hyperlipidaemia Wilson’s disease
Other^§^	Human immunodeficiency virus (HIV) Hepatitis B (HBV) and C (HCV) virus Environmental hepatotoxins: Phosphorus; Petrochemicals; Toxic mushrooms; Organic solvents	Inflammatory bowel disease Small-bowel diverticulosis with bacterial overgrowth Bacillus cereus toxins Autoimmune hepatitis

^†^Decreased appetite, malnutrition, and weight loss are common features in many psychiatric disorders, ranging from eating disorders, loss of appetite, and secondary weight loss in depression or lack of nutrition in psychotic disorders due to paranoid delusions. Moreover, some patients with severe eating disorders or severe suicide attempts may require parenteral nutrition. Finally, the prevalence of obesity is higher in patients with psychiatric disorders than in the general population, with some requiring from bariatric surgery

^‡^These agents produce fatty liver or liver inflammation. Valproic acid is a mood stabilizer commonly used in bipolar disorder. Cocaine is highly associated with psychiatric disorders such as drug use disorders, as well as affective, psychotic, or personality disorders. The use of glucocorticoids may sometimes induce manic or depressive states. Patients with psychiatric disorders have higher prevalence of HIV and require the use of antivirals for infection control. The association of fatty liver with amiodarone is strong, whereas its association with valproic acid or calcium-channel blockers is weak. Drug-induced fatty liver may have no sequelae (e.g., cases caused by glucocorticoids) or can result in cirrhosis (e.g., cases caused by methotrexate and amiodarone)

^§^The prevalence of HIV, HCV, and HVB in patients with psychiatric disorders is higher than in the general population. Moreover, some suicidal attempts, although uncommon, may be due to environmental hepatotoxins, such as organic solvents

**Fig. 1:**
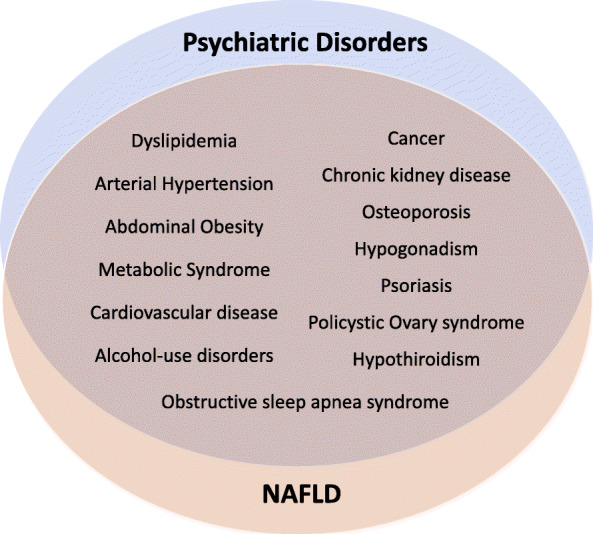
Common medical comorbidities among NAFLD and psychiatric disorders

Ultrasonography plays a key role in the determination of steatosis, and a step-by-step approach is preferred [13]. The precise global incidence of NAFLD is unknown, due to the complex screening required, wide variations in the enrollment of differing populations, racial groups, exclusion criteria for alcohol consumption, study design, and diagnosis methods [14]. However, its prevalence is thought to be on the rise, ranging from 20–30% in Western countries to 8–20% in Africa [15, 16]. There are many common medical comorbidities among NAFLD and most psychiatric disorders, including cardiovascular and endocrine-related diseases such as MetS, diabetes mellitus, dyslipidemia, arterial hypertension, obesity, hypothyroidism, hypogonadism, polycystic ovary syndrome, osteoporosis, obstructive sleep apnea syndrome (OSAS), chronic kidney disease, psoriasis, or cancer [17–20] (Fig. 1). Taking into account its disease burden and growing prevalence, and the neglected relationship of NAFLD with MetS and psychiatric disorders, there is a need to better understand this disease and its commonalities with psychiatric illnesses, in order to develop proper and tailored assessment and test interventions for this prevalent but neglected comorbidity.

## Methods

To gather data on the subject, a search on PubMed with the terms “([Title/Abstract]NAFLD OR [Title/Abstract]NASH) AND psych*” was performed. We also conducted a ScienceDirect search with the terms “NASH, NAFLD, psychiatry.” A total of 92 results were obtained, of whom only those pertaining the specific link of NAFLD, or NASH, and psychiatric disorders, were included, leaving a total of 11 publications. The small number illustrates the sparsity of the literature describing NAFLD in psychiatry. We also explored secondary references found in the initial bibliography. A secondary search of reviews and data on the physiopathology of NAFLD, and on relevant subjects such as general morbidity of psychiatric disorders, was also performed to give the reader an overview on the underlying mechanisms of this disease and to explore possible commonalities not yet investigated.

## Epidemiology and natural history

NAFLD includes two distinct conditions with different prognoses: non-alcoholic fatty liver (NAFL) and non-alcoholic steatohepatitis (NASH). NAFL is defined by steatosis, meaning the presence of lipidic vacuoles in hepatocytes. NASH involves steatosis plus inflammation, with signs of hepatic disease, such as ballooning, and might involve fibrosis [21]. NASH has a higher risk of cirrhosis and hepatocellular carcinoma [22]. NASH carries a high risk of both liver related morbidity and mortality as well as metabolic comorbidities (ten times higher than the general population), cardiovascular disease and mortality (twice higher than the general population), and cancer (particularly bowel and breast cancer) [17]. Around 20% of patients suffering from NASH will develop cirrhosis [16], although it is important to note that hepatocellular carcinoma can occur in the absence of cirrhosis [23], making NASH the most rapidly growing indication for liver transplantation in patients with hepatocellular carcinoma in the USA [24].

In a recent meta-analysis, the pooled population estimated prevalence of NAFLD was 24–25%, although it varied across countries. Currently, it is thought to occur in around 24% in the general population of Europe and the USA, 27% in Asia, 30% in South America, and 32% in the Middle East. In Africa, prevalence estimates are lower, at around 13%. It is worth noting that this data refers to diagnosis made by imaging, mainly through ultrasound measuring hepatic fat, as studies using blood tests (measuring ALT, AST, and platelet count) report notably lower prevalences [7, 8, 16]. The regional NASH prevalence estimates among NAFLD patients with an indication for biopsy were around 63% for Asia, 69% for Europe, and 61% for North America. On the other hand, NASH prevalence estimates among NAFLD patients without an indication for biopsy were 7% in Asia and 30% in North America [16].

Both NAFLD and NASH are more prevalent among males [19]. Gender differences also exist in most psychiatric disorders, such as schizophrenia, which is more common in males, and depression, which is more common in females [25–27]. On the other hand, racial and ethnic variations exist in NAFLD and NASH; in the USA, the prevalence of NAFLD is highest among Hispanic and lowest among Afro-American populations [28].

While the total prevalence of liver disease in patients with psychiatric illness is not fully known [29], the prevalence of MetS in schizophrenic or bipolar patients is remarkably high, ranging from 22 to 42% [30], compared to non-psychiatric control populations (with an estimated prevalence of 15–20%). A recent meta-analysis found patients suffering from severe mental illnesses to have a pooled RR of 1.58 (1.35–1.86) of developing MetS [2]. Hence, people suffering from mental health disorders are thought to have an increased incidence of NAFLD, although data are scant [18]. In this regard, several studies and publications have aimed to evaluate its comorbidity with specific psychiatric disorders.

Most research in NAFLD in psychiatry focuses on affective disorders. In subjects suffering from NASH, Elwing et al. observed a higher rate of lifetime major depression (MDD) and generalized anxiety disorders (GAD) as compared to the control population. Comorbid anxiety in patients is linked to more advanced liver histological abnormalities [31]. In patients with NAFLD, there is also evidence pointing for more severe hepatic ballooning when they have MDD [32]. It is important to note that these studies examined affective disorders in liver disease patients. The other way around, examining depressed patients, Weinstein et al. found higher prevalence rates and severity of NASH compared to other chronic liver diseases [33]. Two studies among bipolar patients also indicated a higher prevalence of chart-reported NAFLD and NASH [34, 35], pointing to risks and mechanisms beyond lifestyle or drug use, although no sustained explanatory theory has yet been suggested. Unhealthy lifestyle habits are disproportionally present in affective disorders, where inflammatory and metabolic disorders are similarly frequently found.

A higher prevalence of NAFLD in schizophrenia is likely [35, 36]. Yan and colleagues, in a large cross-sectional study comparing young males with schizophrenia with general young males, found a significantly higher prevalence of NAFLD of up to 49.5% in the study group compared to 20.1% in the control group. They found the factors most correlated with NAFLD were triglyceride levels, BMI, medication combinations, drug dosage, and negative factor scores on the PANSS [37].

Anorexia nervosa is also associated with significant liver complications, as fasting can lead to steatosis [38]. However, we found no study assessing the specific prevalence of NAFLD in these patients. Regarding other psychiatric disorder populations, such as those with anxiety, OCD, or PTSD, we are not aware of specific studies examining the comorbidity with NAFLD/NASH.

## Pathophysiology of NAFLD/NASH

**Fig. 2:**
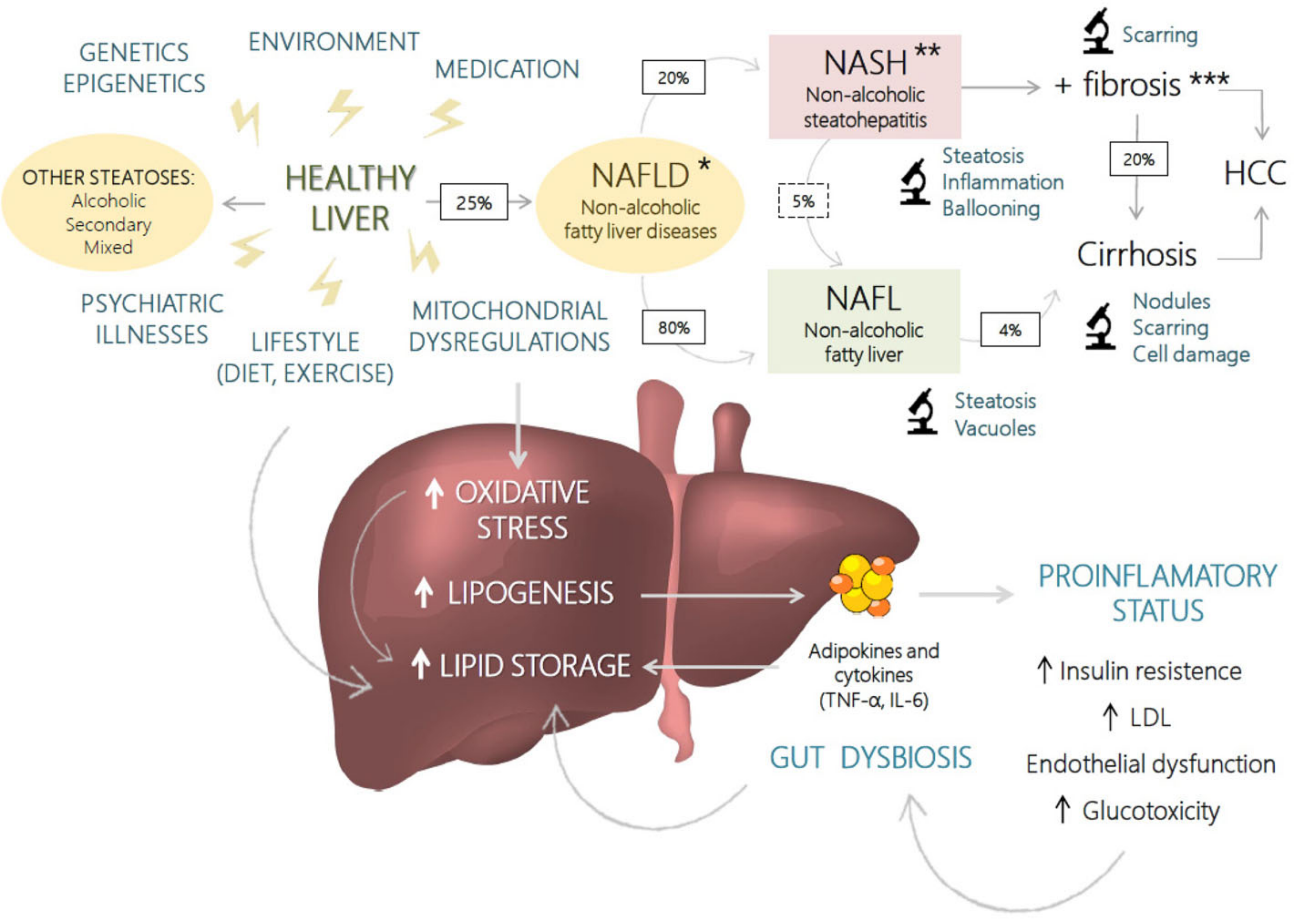
Pathophysiology and development of non-alcoholic fatty liver disease, with associated risk factors. Histopathological lesions of NAFLD have been graded and staged regarding steatosis, steatohepatitis and fibrosis ([10], adapted from Brunt et al.). *Grading for steatosis. According to percentage of affected hepatocytes: grade 1 (< 33%), grade 2 (33–66%), and grade 3 (> 66%). **Grading for steatohepatitis: grade 1 or mild (macrovesicular steatosis, up to 66% of lobules; occasional ballooning; mainly scattered acute lobular inflammation; none or mild portal inflammation), grade 2 or moderate (mixed macrovesicular and microvesicular steatosis; obvious ballooning; possible mild chronic lobular inflammation; pericellular fibrosis; mild to moderate portal inflammation), and grade 3 or severe (> 66% of lobules affected by steatosis; marked ballooning; scattered acute and chronic lobular inflammation; perisinusoidal fibrosis; mild to moderate portal inflammation). ***Staging for fibrosis: stage 1 (focal or extensive, perivenular, perisinusoidal, or pericellular), stage 2 (with added focal or extensive periportal fibrosis), stage 3 (focal or extensive bridging fibrosis), and stage 4 (cirrhosis). HCC, hepatocellular carcinoma; TNF-α, tumor necrosis factor α; IL-6, interleukin 6; LDL, low-density lipoprotein

NAFLD, diabetes mellitus type 2, and obesity are thought to share similar risk factors and pathophysiological pathways (Fig. 2). Since the liver is involved in the majority of metabolic routes, NAFLD can be considered a consequence of disturbed metabolism, observed with insulin-resistance and metabolic inflammation [22]. NAFLD develops through the induction of hepatic lipogenesis which, in turn, stimulates dysregulated secretion of proinflammatory cytokines and adipokines; hence, lipid storage in the liver is increased. A complex interaction between genetic, environmental, and epigenetic factors interplays in the genesis of this disease. Importantly, these factors share interesting commonalities with psychiatric diseases. They are not only important to the onset of NAFLD, but also to its progression to NASH, and have an influence in its natural history and severity [22]. A multiple-hit model has been proposed, implying multiple insults acting together on genetically predisposed subjects to induce NAFLD [39]. Similarly, explanatory models for psychiatric disorders are moving towards this conception as well [40].

A number of harmful life habits such as a sedentary life style or an excessive calorie intake have a strong correlation with the development of this disease, as well as an excess intake of saturated fatty acids, polyunsaturated omega 6 fatty acids, and industrial fructose [22]. Low levels of physical activity, and poor diet quality including increased emotional eating (eating in response to negative emotions), have been linked to depressive symptoms [41]. However, unhealthy lifestyles and obesity are not the sole factors in NAFLD genesis, as a relevant percentage (25–30%) of individuals with obesity might present with a lower risk of these alterations (the so-called healthy obesity), whereas there is a subset of non-obese individuals that can suffer from MetS [42]. However, it is relevant to note that healthy obesity still involves a higher risk of developing NAFLD than normal weight [43].

In psychiatric patients, the effects of medication also need to be taken into account, as psychiatric treatments may have specific side effects on the liver. They also contribute greatly to weight gain, and the MetS itself [29]. Moreover, there seems to be an inherent risk of developing MetS in relation to psychiatric illness that is also present in non-treated individuals [2]. Diabetes and insulin resistance have also been associated with an increased risk of depression and anxiety [44]. Recent publications point to depression as an independent risk factor for obesity [45], and this relationship might be bi-directional [46, 47]. Alterations in cortisol, which are more frequent in psychiatric patients, can also promote insulin resistance, and pro-inflammatory cytokines like tumor necrosis factor-α and interleukin-6 are involved in depressive disorders and NAFLD/NASH [48].

## Common factors

### Genetics

Genetic, epidemiological, and twin studies show evidence of moderate heritability of hepatic fat accumulation, ranging from 30 to 70% [49, 50] (Table 2). Genome-wide association studies (GWAS), candidate gene studies, and epigenetic studies have so far discovered a few genetic loci and involved proteins that play an important role in the regulation of lipid metabolism, inflammation, insulin signaling, oxidative stress, and fibrogenesis [51].

**Table 2:** Summarizes the common factors found to have a relevance in the pathogenesis of both NAFLD and psychiatric disorders

Findings		Normal function	NAFLD	Psychiatric disorders
Genetic	PNPLA3 (adiponutrin) polymorphisms	- Hydrolase activity on triglycerides and retinyl esters - In pituitary: regulation of glucose and fatty acid homeostasis, appetite and energy expenditure	Linked to pathogenesis	Bipolar disorder - Unknown mechanism - Probably linked to inflammation and oxidative stress
	miR-34a	- Hepatic lipogenesis & lipid secretion - Neurodevelopment & synaptogenesis	NAFLD progression & heritability	Bipolar disorder - Elevated in diagnosed of BD - Decreases in response to lithium
Mitochondria, inflammation and oxidative stress	Altered mitochondrial metabolism	- Protection against fatty acid accumulation - Energy production	Excessive oxidative species are linked to hepatic inflammation, accumulation of fatty tissue and progression of NASH	Linked to: - Pathogenesis: neuroinflammation, dysregulation of brain energy generation & dysfunction in stress response mechanisms - Progression & poorer outcomes In several disorders: - Bipolar disorder** - Depression - PTSD - Psychosis & schizophrenia - Autism
Microbiota	Gut dysbiosis	- Digestion of nutrients - Production of vitamin K & B - Maintenance of the intestinal mucosa - Immune barrier effect	- Lipid accumulation in the liver - Increased absorption of disaccharides - Accelerated hepatic lipogenesis - Inflammation and steatosis	ADHD, autism, depression, dementia - Inflammatory dysregulation mediated by bacterial products - Probiotics as suggested therapies
Psychological factors, lifestyle, exercise and diet	Personality traits	Enhancing adequate lifestyles	- Weight gain and fatty tissue proliferation - Dysregulation in immune response	Nonadaptive traits - High neuroticism - Low conscientiousness
	Exercise	Protective effects against inflammation	Low activity linked to - Weight gain and fat accumulation in liver - Impaired glucose metabolism - Upregulation of immune response, inflammation and fibrosis	Poorer mental health - Low levels of activity linked to higher risk of depression
	Impaired glucose metabolism and DM2	Regulating levels of exertion and fatigue during exercise	- Weigh gain - Accumulation of fatty tissue in liver - Increased lipogenesis - Increased ROS and lipid metabolism by-products - Upregulation of inflammation	Unhealthy lifestyles Link to higher risk of depression
	Obesity	Normal diet secures energy intake and essential nutrients	NAFLD - Fatty tissue proliferation - Inflammation and oxidative stress	- Unhealthy lifestyles - Medication adverse events - Higher risk of depression

Among them, patatin-like phospholipase domain-containing protein 3 (PNPLA3), also known as adiponutrin, variation 148M, is the most robust and replicated in different studies, including GWAS [52] and meta-analysis [53]. The protein adiponutrin has a hydrolase activity on triglycerides and retinyl esters. We did not find genetic studies assessing the potential links between NAFLD and psychiatric disorders. Kenneson and Funderburck, however, found individuals with the recessive variant of the PNPLA3 genotype (MM) had an adjusted odds ratio for bipolar disorder of about 4.6 compared to individuals with either the IM or II variants [54]. They proposed an explanation based on the relationship between inflammation, NAFLD, and bipolar disorder. Adiponutrin is also expressed in the pituitary, where it appears to be involved in the regulation of energy homeostasis [55]. In this regard, adiponutrin would play a role in regulating glucose and fatty acid homeostasis, appetite control, energy expenditure, and the response to starvation through its concentration in this gland. There is also evidence of dysfunction of energy regulation and the HPA axis in bipolar patients, suggesting similar pathways for metabolic dysregulation [56].

There is also a growing interest in the relationship between NAFLD and microRNA (miRNAs), which are short (19–23 nucleotides) non-coding RNA molecules that regulate messenger RNA degradation or translation, thereby modulating the expression of entire sets of genes and pathways [51]. MiRNAs are important for signaling stress and distress between cells, and these pathways have been positively linked to NAFLD progression [57, 58] and heritability [59]. A distinct profile of miRNAs for NAFLD might be useful for its diagnosis [60]. An increasing body of evidence is delving into their role in psychiatric disorders [61]. Although there are no studies assessing the specific and common roles of microRNA in NAFLD and psychiatric disorders, its importance in terms of inflammation regulation hints to the involvement of common pathways. For example, miR-34a regulates hepatic lipogenesis and lipid secretion, and is elevated in serum in patients with NAFLD [59]. In bipolar patients, this same miRNA is also elevated, and it has been hypothesized that it might be an important link between diverse genetic risk factors and their translation into the disease, regulating fundamental molecular networks for neurodevelopment and synaptogenesis. Moreover, miR-34a concentrations change in response to treatment, decreasing in response to lithium [61]. However, it is important to bear in mind that both NAFLD and psychiatric disorders are complex, polygenic diseases, and these findings only hint to common pathways, but there is still a lack of adequate powered studies in this regard.

### Mitochondria, inflammation, and oxidative stress

As the site where free fatty acid oxidation takes place, mitochondria play an important role in protecting against fatty acid accumulation. Consequently, impairment of their metabolism can lead to an excess of oxidative species that is associated with the development of NASH [62]. Several mutations in this process have been linked to NAFLD and NASH [51]. Mitochondria, inflammation, and oxidative stress interplay in a complex manner. Oxidative stress is closely linked to inflammatory dysregulation, which, as noted before, is a key feature for the development of NASH from NAFLD and hence could influence on the severity of the disease [22]. In fact, patients with steatohepatitis have ultrastructural mitochondrial lesions, which are absent in most patients with simple steatosis [62, 63]. This mitochondrial injury may lead patients with steatohepatitis to slowly resynthesize ATP, causing acute hepatic ATP depletion [64].

In parallel, mitochondria, inflammation, and oxidative stress are also important links in the pathogenesis of psychiatric disorders, especially mood disorders [65–68]. Current theories propose a model based on neuroinflammation and dysregulation of brain energy generation, involving dysfunction in stress-response mechanisms [69]. Recent evidence points to mitochondria as one of the main loci for the pathogenesis of bipolar disorder [70]. An increasing number of agents capable of enhancing antioxidant defenses or mitochondrial functioning have recently been studied for the treatment of mood disorders, which could also be worth considering as adjuvant therapy to current pharmacological treatments for NAFLD [71]. Other disorders, such as PTSD [72], psychosis [73, 74], schizophrenia [75], depression [76–78], and autism [79], have also been linked to oxidative stress and mitochondrial dysregulation. These alterations not only are linked to the disorder pathogenesis itself, but also to progression of the disorder and poorer outcomes, such as worse cognition in schizophrenia [80].

Thyroid alterations, known to be involved in the regulation of metabolism and inflammation, have been linked to psychiatric disorders [81, 82], and also play an important role in the accumulation of lipid in the liver, potentially driving to NAFLD independently [83, 84].

### Microbiota

Gut dysbiosis can contribute to the accumulation of fat in the liver and the pathogenesis of NAFLD and NASH, promoting the intestinal absorption of monosaccharides, and accelerating hepatic lipogenesis [85]. Moreover, bacteria-derived products can induce inflammation of adipose tissue, hepatic steatosis, and hepatic inflammation [86]. This has led to research targeting microbiota as therapeutic target for these diseases [87], with some probiotics already in the testing phase [88].

The relationship between microbiota and psychiatric illnesses is a hot topic [89], as microbiota has been linked to a wide range of psychiatric disorders, from ADHD and depression to autism and dementia. The main theories explaining this relationship point to inflammatory dysregulation mediated by bacterial products [90, 91]. Some probiotics are also being tested as therapies, although the evidence remains scant [92, 93]. However, we found no study assessing common microbiota alterations in NAFLD and psychiatric illnesses.

### Psychological factors, diet, and exercise

As mentioned, unhealthy lifestyle habits play a central role in the pathogenesis of NAFLD. In this regard, psychological factors that influence these behaviors are key elements. Stewart et al. [94] found that low conscientiousness and high neuroticism were associated with weight gain and higher risk of NAFLD, while high physical activity has been found to exert a protective effect [95, 96].

The rating of perceived exertion is defined by sensations of effort, constraints, discomfort, and fatigue felt by a person when exercising or engaging in physical activity and has an influence in maintaining such activity. Interestingly, in NAFLD, high levels of perceived exertion have been linked to metabolic factors, such as glucose levels, suggesting an impaired glucose metabolism as a maintenance factor of unhealthy life styles. This association was not found in other hepatic illnesses, such as hepatitis C [97].

Poor nutrition is strongly linked to NAFLD. Excessive energy intake, particularly from complex carbohydrates, as well as fructose consumption, saturated fats, and industrialized food products are linked to a dysregulated metabolism and to higher risk for the development of NAFLD [98]. These same dietary factors have been linked to poor mental health, as they influence in several pathways related to psychiatric disorders, including inflammation, oxidative stress, the gut microbiome, epigenetic modifications, and neuroplasticity [99].

## Treatment

The best and most effective treatment for NAFLD and NASH remains a change in lifestyle, promoting a better diet, weight loss, and physical activity [7] (Table 3). In the early stages, a weight loss of 5–8% and a healthy diet might be sufficient. In a recent systematic review, NAFLD/NASH was even proposed as a cognitive-behavioral disease, as lifestyle changes are its most effective management strategy [100]. However, in more advanced stages of liver disease, pharmacological treatment, mainly insulin sensitizers, and more aggressive approaches such as bariatric surgery, might be needed [86]. Exercise, healthy diet, and weight loss also substantially improve the outcome of several psychiatric illnesses, improving factors such as cognitive functioning, negative symptoms, depression or anxiety, and have a beneficial impact on common pathways like inflammatory regulation [99, 101].

**Table 3:** Take-home ideas about non-alcoholic fatty liver disease related to psychiatric illnesses

SUMMARY PANEL	
NAFLD = strongly related to MetS NASH = steatosis + inflammation	
NAFLD and NASH are core elements driving metabolic diseases that are often neglected. NASH implies a high risk of progression to cirrhosis and hepatocellular carcinoma.	
Psychiatric conditions and NAFLD are bidirectionally related. Rates of NAFLD and NASH in psychiatric patients are high. 60% of the excess mortality in psychiatric patients is due to physical comorbidities.	
Some common factors between psychiatric and metabolic disorders are: genetic (adiponutrin, microRNA), mitochondrial and oxidative stress dysregulations, dysbiosis, psychological factors and lifestyle (diet and exercise).	
Possible treatments include changes in lifestyle, insulin sensitizers or statins.	
Diagnosis involves imaging and histology; therefore, widespread screening is difficult. Some questionnaires and biological markers are being investigated in order to make diagnosis easier and less invasive.	
Being aware of covert hepatic disorders and achieving an early diagnosis and adequate treatment could potentially benefit psychiatric patients in terms of prognosis and quality of life.	

*Abbreviations*: *NAFLD* non-alcoholic fatty liver disease, *MetS* metabolic syndrome, *NASH* non-alcoholic steatohepatitis

Among people with psychiatric disorders, even though having NAFLD does not necessarily mean impairment of hepatic function, it is important to bear in mind that a vast number of psychiatric drugs are hepatically metabolized, and their half-life, secondary effects profile, and metabolism should be accounted for [29]. As noted before, some medications are much more associated with the MetS than others.

Some therapeutic approaches are proposed to target the metabolic imbalance that underlies NAFLD. They might also prove beneficial for psychiatric disorders, based on the common mechanisms mentioned before. For example, new insulin sensitizers targeting mitochondrial pathways are being tested for this purpose. They could improve the metabolic pathways leading to diabetes mellitus type 2, inflammation, and oxidative stress and hence could be of help in both entities [102]. Some insulin sensitizers may have value for psychiatric disorders as well, although data remain preliminary [103]. To target oxidative stress, vitamin E has recently been tested as a treatment with promising results [104]. Based on the correlation between poor life style and depressive symptoms, as mentioned before, antidepressants might also be considered treatment for NAFLD, although the potential secondary side effects affecting metabolism of some agents should be taken into account [105, 106].Statins are also interesting drugs for treating both NAFLD and psychiatric illnesses. They have a role in diminishing free cholesterol and have been found to protect against histological damage [107]. As they have anti-inflammatory and anti-oxidative effects, they have also been proposed as adjuvant therapies for a number of psychiatric disorders [108]. Another interesting example of common pathways and treatment can be found in the role of cannabinoid 1 receptor, which modulates the hepatic energy state and food intake, and could play a role in the development of hepatic steatosis [109]. The role of cannabinoid receptors in the development of psychiatric disorders is being widely studied as well, although the evidence remains scant and caution is advised [110].

## Diagnosis

As mentioned before, NAFLD and NASH have a high prevalence in the general population, but this is even higher in psychiatric patients. There is a need for NAFLD and NASH to be taken into account when assessing such patients, and their specific impact needs to be further investigated. However, as NAFLD is frequently asymptomatic, and is not detected with current psychiatric medical screening protocols, it is likely grossly underdiagnosed and disregarded. On the other hand, the current criteria are based on ruling out other possible causes, whereas, based on the knowledge on the pathophysiology of the disease, new nomenclature based on specific causes has been proposed, aiming for better and more precise definition and diagnosis [111]. The notion of metabolic dysfunction-associated fatty liver disease (MAFLD) has recently been suggested, involving positive criteria that relate to the probable metabolic-inflammation impairment that underlies this pathology [11].

Diagnosis is currently based on imaging and histology. A widely used surrogate index, the fatty liver index (FLI) [112], might be useful in detecting steatosic changes, but cannot be used for the diagnosis of NASH. To consider a diagnosis of NAFLD, and refer a person for imaging (liver ultrasound), the patient should present with known risk factors, symptoms of fatigue, abdominal pain, or show an abnormal liver function in the liver test. NAFLD diagnosis requires either imaging or histologic demonstration of more than 5% hepatic steatosis in the absence of excessive alcohol consumption. Other possible causes of steatosis should also be ruled out, such as hepatitis C infection or hypothyroidism [105]. In contrast, a NASH diagnosis requires a liver biopsy with histologic examination, which is not feasible, cost-effective, or necessary in every patient with NAFLD. These methods can be invasive and are difficult to implement in the general population for screening purposes. In this regard, recent advances in ultrasonography techniques might allow for rapid, non-invasive, and cost-effective alternative methods, and some instruments such as the ultrasound fatty liver indicator (US-FLI) have proven to yield a high predictive value for NASH diagnosis [113]. Currently, biological markers such as the FLI are only considered appropriate for large epidemiological studies [11].

There is also a lack of reliable biomarkers [17], although there are some efforts made in this direction, based on mechanisms mentioned before, like inflammatory profiles [114] or miRNAs [60], that could help in aiding an early diagnosis and more closely monitoring the progression of the disease. Some algorithms based on non-invasive methods, and aiming for wider screening, are proposed, as the global awareness of this disease is increasing [19]. Non-invasive scoring systems can estimate the degree of fibrosis without biopsy, helping to assess the risk of progression to NASH and cirrhosis. The Fibrosis-4 (FIB-4) Index, incorporating platelet count, age, AST, and ALT, is the most commonly used and has been validated for NAFLD and NASH [115, 116].

Some questionnaires are also being validated for monitoring impact and progression, like the Liver Disease Questionnaire [117]. Nevertheless, greater awareness and suspicion of the disorder in at-risk cohorts should prompt further investigation. In research, adequate and standardized measures for non-invasive diagnosis and measuring of progression should be implemented, in order to increase the external validity of the results and enhance our knowledge of the real incidence and prevalence of these diseases.

**Fig. 3:**
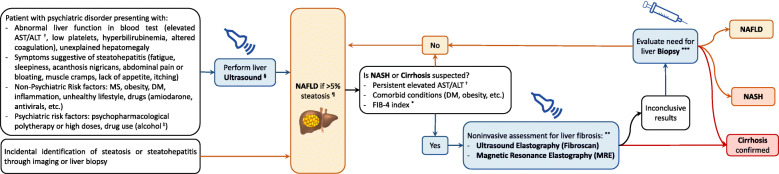
Diagnostic algorithm for non-alcoholic fatty liver disease and non-alcoholic steatohepatitis in psychiatric populations. † Elevated AST/ALT levels should be considered from 1.5 times the upper limit of normal values. However, normal levels do not preclude a diagnosis of NAFLD. ‡ The diagnosis of NAFLD requires the exclusion of alcohol abuse. However, alcohol use is the most common cause of induced hepatic fibrosis and cirrhosis and it should be considered particularly in psychiatric populations. In the case of high alcohol intake, non-invasive assessments for liver fibrosis (commonly an ultrasound elastography) should be performed. § Ultra-sound misses around 20% of steatosis diagnoses. Steatosis may be detected on non-contrast CT, but due to similarity to or lower sensitivity than ultrasound, exposure to radiation, and potential for misdiagnosis, it is less useful than ultrasound as a screening test. Magnetic resonance imaging (MRI) is the most sensitive modality for the evaluation of hepatic steatosis (with 92%-100% sensitivity, 92%-97% specificity, and the ability to reliably detect as little as 3% steatosis) but is significantly more costly than ultrasound. None of these imaging modalities can differentiate NAFLD from NASH, and they have limited ability to discern those patients with advanced fibrosis. ¶ The distinction of NASH from simple steatosis is key. Steatosis due to secondary causes, such as viruses, autoimmune responses, metabolic or hereditary factors, and drugs or toxins, should be ruled out (Table 1). However, the rising prevalence of NAFLD makes its coexistence with other chronic liver diseases quite possible. Therefore, a positive diagnosis rather that a diagnosis based on exclusion of concomitant diseases has been proposed10. Over the last years, the ability to identify NAFLD and to estimate steatofibrosis with ultrasound-based techniques (semi-quantitative, quantitative, elastographic, and contrast-enhanced) has undergone tremendous progress. However, it is still difficult to capture the inflammatory component of NASH with ultrasound-assisted techniques112. * The FIB-4 index is a simple formula based on age, ALT, AST, and platelet count that predicts fibrosis and has been validated in NAFLD and NASH. However, patients in the indeterminate cut-off values require additional testing to exclude fibrosis. ** Non-invasive imaging–based evaluation for fibrosis primarily relies on measuring elastic shear wave propagation through the liver parenchyma, with stiffer fibrotic tissue propagating waves faster. The best-validated methods are transient elastography using ultrasound, such as FibroScan, which has a sensitivity of 85% for detecting advanced fibrosis and 92% for detecting cirrhosis, and magnetic resonance, which provides a quantitative estimation of liver fat, but it is comparatively expensive, has limited availability, is time-consuming, and requires special software. *** Liver biopsy, although typically well tolerated, can be painful and can carry morbidity such as bleeding, infection, bile leak, damage to other organs, and rare mortality risk (<0.01%). Abbreviations: DM: diabetes mellitus; FIB-4: fibrosis 4 index; MRE: Magnetic Resonance Elastography; MetS: metabolic syndrome; NAFLD: nonalcoholic fatty liver disease; NASH: non-alcoholic steatohepatitis.

In Fig. 3, we propose a practical diagnostic algorithm for NAFLD and NASH tailored for psychiatric populations, based on clinical experience and the American Association for the Study of Liver Diseases (AASLD) guidelines [8, 19].

## Conclusion

**Fig. 4:**
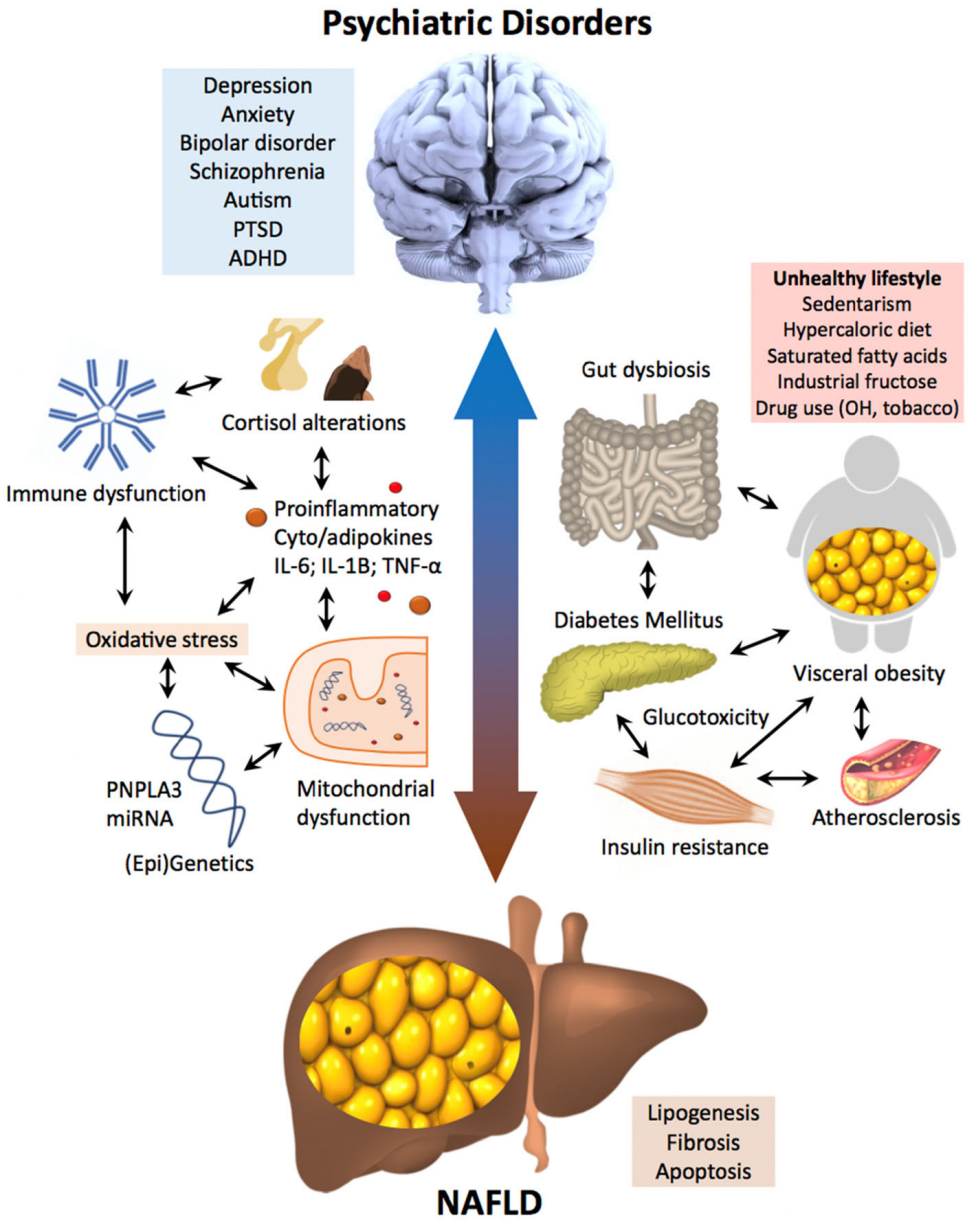
NAFLD and psychiatric disorders. Bidirectional pathophysiological relations between NAFLD and psychiatric disorders. NAFLD and psychiatric disorders share many common pathophysiological pathways that point to common underlying mechanisms. NAFLD is part of a complex system of mental and organic diseases with a common pathogenesis between genetic, environmental, and epigenetic factors based on dysregulation of inflammation, redox pathways, and mitochondrial biogenesis. Abbreviations: ADHD, attention deficit and hyperactivity disorder; miRNA, micro RNAs; NAFLD, non-alcoholic fatty liver disease; OH, alcohol; PNPLA3, patatin-like phospholipase domain-containing protein 3; PTSD, post-traumatic stress disorder

Prevalence studies show higher rates of NAFLD and NASH among people suffering from psychiatric disorders, suggesting common risk factors and pathophysiological links between these entities, with the MetS at their core. Although few studies have been performed to assess this relationship, common factors such as genetics, mitochondrial dysregulation, and dysbiosis have been found, strengthening this theory. NAFLD might be part of a complex system of psychiatric and non-communicable medical disorders with a common pathogenesis based on dysregulation of inflammation, redox pathways, and mitochondrial biogenesis (Fig. 4).

There is a knowledge gap that needs to be filled in order to proper understand the relationship of psychiatric illnesses with NAFLD and its impact. Future research should target the common underlying risk factors and biological pathways. Although some treatments have been found and seem promising, their compound effectiveness is yet to be evaluated. Also, new and more efficient screening tools, tailored for psychiatric patients, who seem to be at a higher risk of suffering this disease, need to be developed in order to assess the real impact of this disease. Clinicians should be aware of this entity, as its presentation might be asymptomatic at onset, but an early diagnosis could yield benefits in terms of prognosis and quality of life.

## Data Availability

Data sharing is not applicable to this article as no datasets were generated or analyzed during the current study.

## Abbreviations

NAFLD: Non-alcoholic fatty liver disease
NASH: Non-alcoholic steatohepatitis
MetS: Metabolic syndrome
FLI: Fatty liver index
OSAS: Obstructive sleep apnea syndrome
NAFL: Non-alcoholic fatty liver
MDD: Major depressive disorder
GAD: Generalized anxiety disorder
PTSD: Post-traumatic stress disorder
OCD: Obsessive compulsive disorder
GWAS: Genome-wide association studies
PNPLA3: Patatin-like phospholipase domain-containing protein 3
miRNA: Micro RNA
ADHD: Attention deficit and hyperactivity disorder
FIB-4: Fibrosis-4 Index
AASDL: American Association for the Study of Liver Diseases
